# Effects of a brief online emotion word learning task on negative emotion differentiation, emotional self-efficacy, and prospective distress: Preliminary findings

**DOI:** 10.1371/journal.pone.0299540

**Published:** 2024-02-28

**Authors:** Lindsey M. Matt, T. H. Stanley Seah, Karin G. Coifman

**Affiliations:** 1 Lyra Health, Burlingame, California, United States of America; 2 Department of Psychiatry, University of Pittsburgh, Pittsburgh, Pennsylvania, United States of America; 3 Department of Psychological Sciences, Kent State University, Kent, Ohio, United States of America; URV: Universitat Rovira i Virgili, SPAIN

## Abstract

**Background:**

Disruptions in emotion processing are common across psychological disorders. Research suggests that emotion differentiation (ED; specificity in language used to characterize one’s emotional experience) and emotional self-efficacy (ESE; perceived ability to understand and manage one’s emotions) are important transdiagnostic factors associated with various psychological benefits. Whether ED and ESE can be improved in adults remains largely unclear.

**Methods:**

Using a longitudinal prospective design, we tested a brief online training targeting emotion word knowledge (vs. a control condition) to see if it improved negative ED (NED) and ESE in a college sample. Moreover, we tested if changes in NED or ESE mediated the effects of the training on levels of psychological distress one week and two-months post-intervention.

**Results:**

Findings provided partial support for our hypotheses. Individuals whose ESE increased post-intervention reported lower levels of distress two months later. Moreover, exploratory analyses revealed those who demonstrated greater training engagement experienced increases in NED that in turn predicted lower distress one-week post-intervention. However, there were no direct effects of intervention group on NED or ESE and distress.

**Conclusions:**

These findings highlight the potential of a remotely-administered emotion-language intervention to influence key dimensions of emotion processing and suggest avenues for further refinement. Both NED and ESE may be malleable for some, and that enhancements in ESE may produce long-term psychological benefits.

## Introduction

To date, mounting evidence from decades of research suggests that disruptions in emotion-related processes are common across psychological disorders and linked to the development and maintenance of psychopathology [1]. These deficits can be understood as manifesting on various dimensions, including how individuals understand their emotional experiences and perceive their ability to manage or cope with emotions. Indeed, deficits in awareness and difficulties identifying one’s emotional experience have been robustly associated with elevated symptomatology [2,3]. Moreover, patients often appear to exhibit relatively simplistic or impoverished conceptualizations of emotional experiences (e.g., alexithymia) [4], report highly polarized affective states [5], and view their experience of negative emotions (e.g., anxiety) as aversive or unmanageable [6]. Indeed, avoidance of attention to negative feelings can result in the engagement in maladaptive behaviors (e.g., substance use; avoidance of feared situation) that interfere with mental health treatment and worsen prognosis [7]. As the mental health field continues to move toward transdiagnostic approaches to diagnosis and treatment, efforts to identify specific emotion processes that cut across mental health conditions are crucial.

Past research suggests that emotion differentiation (ED; also termed “granularity”), which is the specificity of the language individuals use to describe and label their emotional experience [8] may be a particularly important component in adaptive emotional processing and a worthy target for treatments [9]. While improving patients’ facility with emotion language (a process inherent in ED) is common amongst psychological treatments, this is only explicit in some treatments, where emphasis on increasing emotion language literacy and facility is emphasized (e.g., emotion-focused therapy) [10]. Further, relatively little is known as to under what conditions or processes ED ability can be enhanced. The present research aims to address this gap in the literature by examining the effects of an emotion word learning task (relative to a control condition) on ED ability assessed in daily life.

The field has witnessed a surge in ED research in recent years, with most studies reporting significant associations with clinically-relevant affective and behavioral outcomes [9,11]. Specifically, higher levels of negative ED (or NED) have been associated with use of adaptive emotion regulation strategies [8,12], higher levels of self-reported mindfulness and less emotional lability [13], as well as better social functioning [14,15]. Importantly, some studies have demonstrated that healthy individuals tend to exhibit greater levels of NED compared to clinical samples (e.g., depression [16]; borderline personality disorder [17]; autism spectrum disorder [18]), with NED associated with greater wellbeing [19], suggesting that it could be generally protective against psychopathology. More importantly, studies have consistently found that NED predicts less engagement in a broad range of maladaptive behaviors commonly enacted to reduce distress [20], even in times of heightened stress [21].

Indeed, recent meta-analyses revealed consistent protective effects suggesting reduced reliance on such risk behaviors as substance use, binge eating, aggression, and social avoidance for individuals with higher NED, regardless of diagnostic status [11]. Hence, for individuals with clinically heightened levels of distress, higher NED may be protective. For example, higher NED predicts fewer urges and acts of non-suicidal self-injury in borderline personality disorder [22] and less social avoidance in individuals diagnosed with social anxiety disorder and individuals with sub-threshold social anxiety [23]. Similarly, among non-clinical samples, NED appears to be protective against binge drinking [24], aggression [25], and impulsive behaviors [26]. Notably, even in highly challenging contexts, NED appears protective against risk behaviors (e.g., substance use and binge-eating) during the COVID-19 pandemic [27] and may also facilitate adaptive health behaviors during chronic illness [28,29]. Taken together, these findings highlight ED as a potential transdiagnostic factor, with higher levels conferring clear psychological benefits, and lower levels contributing to the development and maintenance of a variety of psychological conditions.

Several mechanisms may underlie the benefits of NED. Certainly, there are clear associations between NED and greater reports of adaptive emotion regulation strategy use, suggesting that the ability to specify emotional experience provides valuable insight and information to make better choices [9,30]. A complementary line of research has begun to explore how precise emotion labeling, a process underlying ED, may facilitate implicit emotion regulatory action. For instance, Kircanski and colleagues [31] tested if emotion labeling could enhance traditional exposure therapy in a group of spider-phobic individuals. In one of four conditions, a group was explicitly instructed to employ affect labeling to describe their negative feelings and worries about the spider during exposure. Affect labeling was found to be more effective than the other three conditions employing conventional emotion regulation strategies (i.e., reappraisal, distraction) and controls (i.e., exposure alone) in reducing emotional arousal (indexed via skin conductance), and more effective than distraction in reducing behavioral avoidance when viewing the spider one week later. These findings are largely consistent with a recent meta-analysis of neuroimaging research suggesting that just the act of conceptualizing emotional experience by activating emotion concepts can reduce reactivity in the amygdala [32].

### Enhancing emotion differentiation

Given its broad potential impact on mental health, we must then wonder what processes could make ED more or less malleable. A recent investigation testing an emotion knowledge intervention demonstrated small benefits to participants by increasing NED during a lab task. In this study by Vedernikova and colleagues [33], participants received five days of training on emotion concepts and were encouraged to then apply them to their own lives during the training. After, they completed a hypothetical scenario task in which ED was derived. The experimental condition showed small but significantly higher NED than controls. Importantly, however, ED was measured from a lab task and not daily life experiences as is most commonly done. Other research by Van der Gucht et al. [34] explored enhancing NED through mindfulness-based intervention that included guided group meditation as well as informal practices focused on increasing in the moment awareness of experiences and sensations. They found that individuals exposed to an eight-week mindfulness-based stress reduction course showed improvements in NED both at post-task and four months later, though these results became non-significant when controlling for mean levels of negative affect. Taken together, these findings suggest that NED is malleable and that these changes could be influenced by targeted intervention.

More broadly, many common psychotherapies include explicit components that emphasize emotion concept and language learning such as Dialectical Behavior Therapy [35,36] and Coping Cat [37]. These treatments have been effective in reducing self-destructive behavior in adults [38] and distress/anxiety in children [39] compared to treatment-as-usual. Basic components include education about the function of emotions and practicing self-labeling of feelings. Randomized control trials of emotion-focused therapies (e.g. person-centered, Gestalt) which encourage explicit attendance to and use of emotion as a source of key information suggest that these treatments are also effective in managing depression equivalent to or better than other approaches [40]. A more recent study also found that an emotion regulation task, which included psychoeducation on emotions, including how to attend to and label emotions in oneself, and the link between emotions, behaviors, and triggers, was associated with less engagement in risk behaviors and greater use of emotion regulation strategies in a group of early adolescents [41].

Emotion language learning is also being both assessed and taught more commonly in schools. A theoretically based emotions course that focused on teaching toddlers to identify and label their emotions was shown to increase performance on tests of emotion knowledge and also contributed significantly to the development of emotion regulatory skills [42]. Other programs that teach specific emotion words by associating them with related antecedents and behaviors (i.e. I Can Problem Solve, ICPS [43], Promoting Alternative Thinking Strategies, PATHS [44]; Second Step [45]) are related to increases in prosocial behavior and reductions in behavioral problems [46–48]. Finally, the RULER (Recognizing, Understanding, Labeling, Expressing, and Regulating Emotions) social-emotional learning program has been shown to consistently improve social cohesion in classrooms and also increase engagement and reduce problematic conduct in at-risk children and teens [49,50].

### Emotion differentiation and emotional self-efficacy

The research described above has suggested that processes by which ED may exert benefits and reduce distress is twofold: 1) by informing individuals more precisely of their experience (perhaps facilitating more adaptive responses [30,51] and 2) by implicitly down-regulating arousal associated with the intensity of the experience [52]. However, one key alternative process may be that ED facilitates the individual’s feeling of emotional competence. This *perception* of one’s ability to perceive and understand, as well as to utilize and manage emotions is known as emotional self-efficacy (ESE) [53]. Broadly, self-efficacy has been defined as the belief individuals have that they are capable to produce change with their own actions [54]. While not considered a trait, self-efficacy develops alongside symbolic thought and language in childhood as we come to understand cause and effect relationships and self-awareness [54] and is then bolstered by responsive environments that can be influenced by personal action. When older, self-efficacy continues to grow when individuals engage in successful attempts to control the environment that they then attribute to their own behavior [55].

Low reported levels of domain-general self-efficacy are known to be associated with depression, anxiety, and avoidant behavior [55,56]. Alternately, higher levels of self -efficacy are associated with greater success in therapeutic interventions to manage substance abuse and eating disorders [57]. Moreover, self-efficacy is a key component of self-regulation with those high in self-efficacy more likely to set high goals and, in pursuit of them, modulate their level of effort as they engage in goal-directed behavior and appropriately persist when faced with challenges [58,59]. Importantly, self-efficacy may be best measured within specific domains [60]. As higher levels of self-efficacy are associated with better functioning in their given domain, it follows that higher ESE has been shown to be associated with more positive and less negative concurrent mood [53]. Interestingly, two recent investigations have demonstrated increases in ED just based on repeated demand to report emotions via experience sampling. No intervention was needed, only repeated practice of labeling current emotional experience increased the capacity to specify discrete experiences, improving ED [61,62]. Hence, because practice can drive an increase in self efficacy [63] a link between ESE and ED seems possible and could be a key pathway by which emotion conceptualization can come to reduce levels of distress.

#### Current investigation

To date, a variety of promising interventions have attempted to enhance emotion differentiation alone or in conjunction with other components of mental health. Limitations to recent targeted approaches include a lack of a control group [34], use of a high time-demand intervention [34] and lab-based (vs. daily life) derivation of differentiation [33]. Additionally, the potential role of self-efficacy in the pathway between emotion differentiation and psychological benefits has not yet been explored.

In the current investigation we developed and tested a brief novel online emotion word learning task intended to specifically increase and reinforce participant knowledge of discrete emotion concepts. The development of this task was informed by current therapeutic approaches in work with both adults (DBT) [35] and children (Coping Cat) [37]. We tested the impact of this task (vs. neutral control task) on levels of ED and ESE and whether shifts in either of these constructs would influence changes in psychological distress across time.

We anticipated that exposure to the emotion word learning task would be associated with improvements in NED and potentially ESE, as well as reductions in psychological symptoms and distress. However, we also considered that NED could mediate the association between task condition and psychological symptoms/distress across time. Indeed, it could be that the intervention improves attention and capacity to label emotional experiences, thereby improving psychological health. Yet, it is possible that an alternative mechanism by which this process unfolds was through increases in ESE. Therefore, we considered ESE as an alternative mediator. Further, we explored whether individual differences in levels of engagement with the word learning task influenced the associations described above. Given the relative absence of prior evidence demonstrating that emotion language interventions can impact either process, as well as a marked absence even demonstrating associations between the two constructs, we generally treated all analyses as exploratory.

## Materials and methods

### Transparency and openness

Below we report how we determined our sample size, all data exclusions, all manipulations, and all measures in the study. Study was approved by Kent State University Institutional Review Board in 2014. Data were collected from 2015–2016 and analyzed using R and SPSS. This study’s design and its analysis were not pre-registered.

### Participants

A priori power analyses were initially conducted based on medium effect sizes, which may have overestimated potential expected effect sizes [62]. This a priori estimation did suggest adequate power in a sample of 150 participants. However, the analytic plan changed during the peer review process and subsequent analyses revealed that effects were small and the sample potentially underpowered (see Results). Because post-hoc power estimation has been greatly criticized [64], we instead utilized tests of equivalence to evaluate null effects [65]. These are reported in detail in the results section.

*N* = 150 English-speaking adults from a large Midwestern university were recruited from 2015–2016 via open enrollment from the psychology research subject pool. Participants were required to have an Apple/Android OS smartphone to be eligible for the study. While the sample was unselected, participants displayed higher than average levels of distress compared to a previous undergraduate sample (see Measures section). From this initial sample, 32 individuals were excluded as they failed online accuracy checks (*n* = 2) or did not complete one or both parts of the online task (*n* = 30), leaving a sample of 118 participants. Note that actual sample size used for different analyses varied due to missing data. We noted this important information for each analysis in the results, as well as in Tables 1 and 2.

**Table 1:** Bivariate correlations of baseline and post-task variables (*n* = 118).

Measure	1	2	3	4	5	6	7	8	9	10	11	12
1) Age	---											
2) # Total Diaries	-.003	---										
3) Verbal Intelligence	.12	-.08	---									
4) Baseline Distress	.15	-.06	.18	---								
5) Post-task Distress	.18	-.12	.14	.75**	---							
6) Two-month Distress	.15	-.05	-.04	.59**	.78**	---						
7) Baseline ESES Sum	.06	-.02	.06	-.51**	-.41**	-.31**	---					
8) Post-task ESES Sum	-.05	.21*	.14	-.30**	-.35**	-.30**	.47**	---				
9) Baseline NED	-.18	.10	-.12	-.14	-.26**	-.22*	-.01	-.08	---			
10) Post-task NED	.09	.30**	-.004	.02	-.10	-.09	-.02	.06	.20*	---		
11) Baseline NEI	.16	-.13	.03	.29**	.34**	.31**	-.05	-.19	-.38**	-.17	---	
12) Post-task NEI	.02	-.39**	-.04	.23*	.37**	.39**	-.04	-.24*	-.39**	-.24*	.73**	---
*n*	118	113	117	111	109	94	118	111	110	104	112	105
*M*	20.14	59.55	44.22	0.00	0.01	-0.002	108.06	106.63	.46	.45	1.48	1.52
*SD*	3.23	9.56	7.77	0.94	0.94	0.92	18.65	23.72	.25	.26	0.41	0.47
Range	18–43	29–70	6–56	-1.52–3.80	-1.34–2.68	-1.45–2.61	50–152	31–155	.00-.99	.00-.98	1.00–3.56	1.00–3.23

*Note*. Distress = Aggregated SCL-90/IDAS-II standardized scores; ESES = Emotional Self-Efficacy Scale, NED = Negative Emotion Differentiation, NEI = Negative Emotion Intensity.

*p < .05

**p < .01.

**Table 2:** Comparison of demographics, self-report, and task measures by condition (*n* = 118).

	Neutral	Emotion					
	(n = 60)	(n = 58)	*Χ* ^2^	*p*			
Sex (% female)	80.00	82.76	.15	.700			
Race			.60	.896			
% Caucasian	81.67	84.21					
% African American	8.33	8.77					
% Asian	6.67	3.51					
% Other	3.33	3.51					
Ethnicity (% Hispanic)	5.17	1.75	1.00	.317			
	*Mean (SD)*	*Mean (SD)*	*n*	*t*	*p*	CI Lower	CI Upper
Age (Years)	20.52 (3.90)	19.74 (2.33)	118	1.31	.194	-0.40	1.95
# Total Diaries	58.88 (9.76)	60.21 (9.41)	113	-0.74	.460	-4.91	2.24
Verbal Intelligence	44.33 (6.93)	44.11 (8.63)	117	0.16	.875	-2.63	3.09
Baseline Distress	.13 (1.07)	-.12 (.79)	111	1.39	.166	-0.10	0.60
Post-task Distress	.12 (1.01)	-.11 (.86)	109	1.26	.210	-0.13	0.58
Two-month Distress	.04 (.96)	-.04 (.90)	94	0.41	.680	-0.30	0.46
Baseline ESES Sum	106.32 (18.32)	109.86 (18.99)	118	-1.03	.304	-10.35	3.26
Post-task ESES Sum	104.05 (24.48)	109.35 (22.79)	111	-1.18	.241	-14.21	3.61
Baseline NED	.45 (.25)	.47 (.25)	110	-0.59	.559	-0.12	0.07
Post-task NED	.42 (.24)	.49 (.27)	104	-1.37	.174	-0.17	0.03
Baseline NEI	1.47 (0.35)	1.49 (0.46)	112	-0.23	.817	-0.17	0.14
Post-task NEI	1.53 (0.48)	1.52 (0.47)	105	0.10	.924	-0.17	0.19

*Note*. Distress = Aggregated SCL-90/IDAS-II standardized scores; ESES = Emotional Self-Efficacy Scale, NED = Negative Emotion Differentiation, NEI = Negative Emotion Intensity.

### Procedure

**Fig 1:**
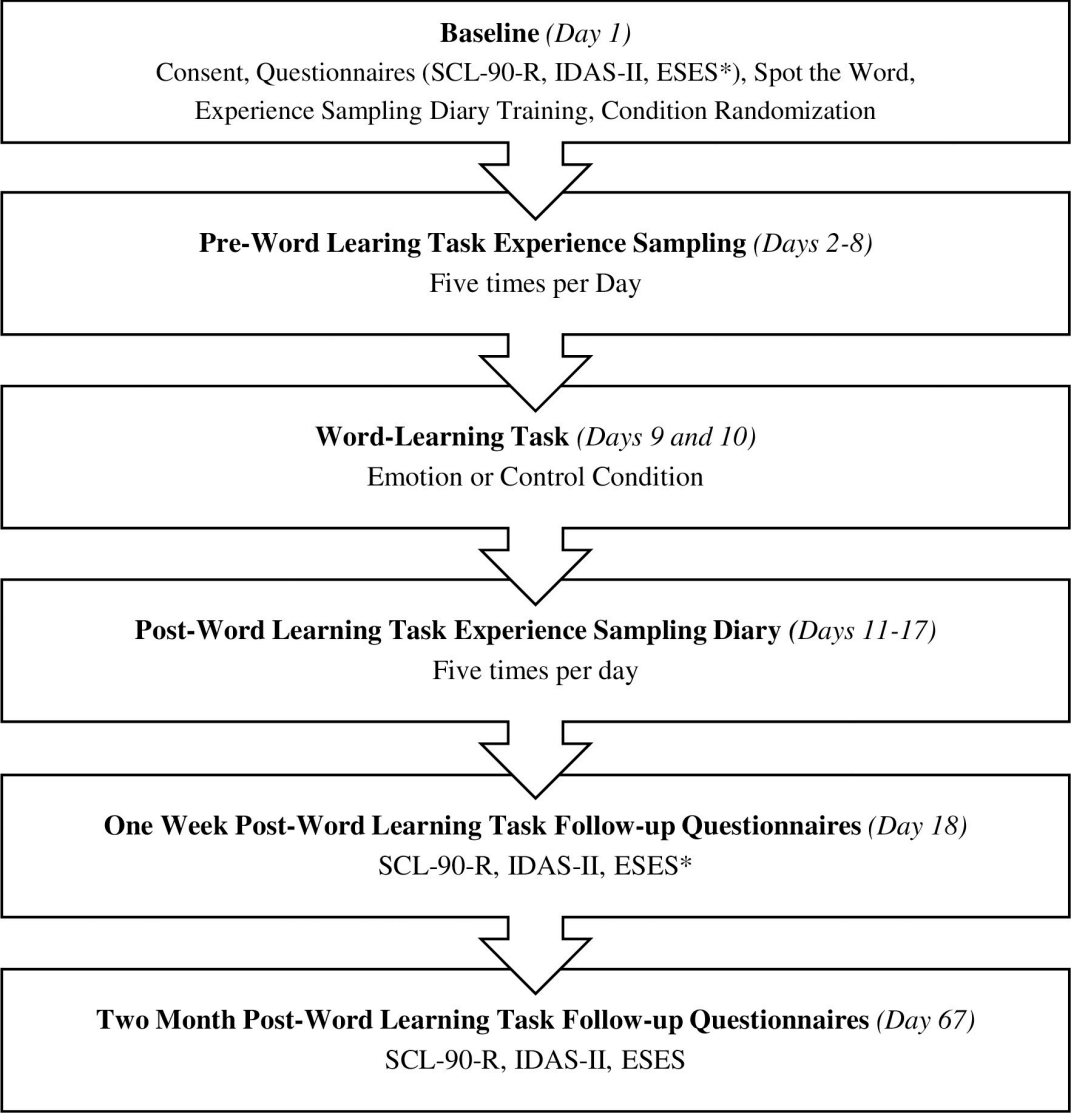
Flowchart of study procedures. SCL-90-R = Symptom Checklist 90-Revised, IDAS-II = Inventory of Depression and Anxiety Symptoms, ESES = Emotional Self-Efficacy Scale.

Study participation took place over approximately two months. Participants completed a baseline lab session where they provided written consent and were assigned a unique study identification number. The consent form detailed risks of study participation including minor risk of mood change in response to the questions being asked or events being remembered. If participants did not wish to answer a question, they were permitted to skip it. They then provided demographic information and reported psychological symptoms, distress, and ESE and were then randomized to one of two word learning task conditions. Verbal intelligence was also estimated at that time. Finally, participants received training on how to respond to experience sampling diary. Following this session, participants engaged in one week of daily experience sampling where they indicated how they were feeling by rating specific emotion words (e.g. anger, sadness) five times a day for seven consecutive days using their smartphones. Afterwards, participants completed an online word learning task across two days, consisting of either emotion (experimental condition) or neutral (control condition) words. Following completion of the online word learning task, participants completed another week of daily experience sampling as above. Emotion word ratings during each phase of experience sampling were used to generate pre- and post-word learning task NED and emotional intensity. Finally, participants completed symptom, distress, and ESE measures again approximately one week and two months after the word learning task. See Fig 1 for flowchart of study procedures. All data collected were labeled by study identification number. Master list of study identification numbers and participant names was secured and able to be accessed only by corresponding author if necessary. Participants and research assistants were blinded to both experimental condition and study hypotheses, and participants were debriefed at the end of their participation.

### Word learning task

Participants were randomly assigned to one of two possible task conditions (emotion vs. neutral) based on their study identification number. The word learning task was sent to participants via e-mail across two sequential days following completion of the first seven days of experience sampling. All parts of the intervention are available online (https://osf.io/sf7mw/).

#### Day one

Participants received an email via Qualtrics asking them to complete a word learning task. They were informed that they would be presented with a series of words and corresponding definitions and asked to study and remember each word as they would be quizzed on them for the next two days. In the experimental condition, fifteen emotion words, including the seven used to derive NED, were presented in random order along with definitions that included detailed information such as the emotion’s function and associated features. For example, fear was described as a feeling that suggests a threat is present, though the danger may be real and present or based on your thoughts. Participants were told that fear may feel like shakiness, sweatiness, and/or jitteriness in the body and that they may respond by seeking shelter, running away, or freezing in place. This presentation was based on discrete emotions theory, which suggests each emotion has a unique antecedent, behavioral output or action [66]. and is modelled after the Emotion Regulation module of Dialectical Behavior Therapy [35] in which individuals are taught about the function of emotions and the role they play in understanding one’s environment.

Techniques from the learning literature were used to help enhance memory of novel information. Following presentation of each word, participants were asked to use a blank space below to write down a related memory that comes to mind. Known as the self-reference effect [67], research has shown that connecting novel information to the self through asking questions such as ‘Does the word describe you?’ results in enhanced memory even when compared to semantic or other-referent encoding strategies [68]. Following presentation of all words, participants completed a randomized-item quiz in which they matched parts of the definitions they learned to multiple choice word options. Use of quizzes in classroom settings is associated with better performance both on exams and in the overall course vs. studying of the material alone [20,69]. Further, multiple choice quizzes (vs. open ended or short answer) offer a concise approach that has been shown to result in enhanced recall of quiz information in the future [70]. Participants were also provided with explicit corrective feedback if they made an incorrect answer selection, as this type of feedback has been shown to significantly increase recall of knowledge, including in assessments of language learning [71].

#### Day two

On the second day, participants received another email via Qualtrics asking them to complete a quiz about the information they read the previous day. This quiz was identical to those seen on day one but presented items in a novel randomized order. Use of repeated testing has been shown to both consolidate learning and enhance recall [72].

#### Control condition

The neutral word condition used the same procedures. However, participants were presented with a variety of words describing household items (i.e. refrigerator, stove) and definitions that included detailed information about their function and history. The presence of a matched neutral word condition allowed for exploration of the unique effects of emotion vs. neutral word learning. These words were chosen as the control condition because we expected participants to demonstrate some variability in familiarity and frequency of contact with different household objects.

### Measures

Tables 1 and 2 provide descriptive information (means, SDs, range) for the whole sample and by task condition respectively.

#### Current symptoms

The Inventory of Depression and Anxiety Symptoms (IDAS-II) [73] is a measure of current symptoms of major depression and anxiety disorders. Given the interest in broad symptoms of psychopathology in the current study, an overall symptom score was created by summing scores from the Dysphoria, Lassitude, Insomnia, Appetite Loss, Appetite Gain, Ill Temper, Panic, Social Anxiety, Traumatic Intrusions, Traumatic Avoidance, and Mania subscales. The symptom score was found to be internally consistent (α = .94) and displayed adequate range (baseline *M* = 126.93, *SD* = 33.45, Range = 68.00–240.00).

#### Current distress

The Symptoms Checklist-90-Revised (SCL-90-R) [74] is a widely-used measure of current symptoms of psychopathology. Here, a combination of the depression, hostility, and anxiety subscales were used to evaluate distress [75]. Scores were averaged to derive an estimate of general distress and showed good internal consistency (α = .93) and range of distress (baseline *M* = .82, *SD* = .54, Range = .03–3.07). Average scores were higher than those in other known undergraduate samples (0.67) [76] but not as high as those seen in clinical populations (1.30–2.00) [77].

#### Aggregate measure of symptoms and distress

Bivariate correlations revealed a significant large positive (*r* = .63 - .78, p < .001) relationship between IDAS-II and SCL-90-R scores at each time point, and thus we chose to standardize and aggregate these variables in our analyses (see Tables 1 and 2).

#### Emotional self-efficacy

The Emotional Self Efficacy Scale (ESES) is a measure of adaptive emotional functioning based on the four-factor model of emotional intelligence [78] and assesses the perceived ability to understand, regulate, and observe emotions in the self and others, and to use emotions to facilitate thought. Items were summed to create a single measure of ESE with good internal consistency (α = .95) and range (see Tables 1 and 2).

#### Verbal intelligence

The Spot the Word task is designed to quickly and accurately estimate premorbid intelligence. It was included in the current study for use as a potential covariate as we sought to isolate the effects of the word-learning task from individual’s baseline vocabulary. It is comprised of sixty pairs of words, in which participants are asked to distinguish between a valid word in the English language (“true” target word) and a non-word foil. A sum score of correct responses was generated and serves as an estimated verbal intelligence quotient [79] (see Tables 1 and 2).

#### Experience sampling diary

During the laboratory session, participants were trained in the use of a mobile phone application (The Personal Analytics Companion; PACO, [80] used to received daily prompts, and were guided through an example prompt to ensure understanding. Individuals were prompted five times within a 14-hour period, with each prompt remaining available for up to 60 minutes.

Each of the prompts assessed current emotional state by asking participants to rate a set of seven negative emotion words (Fear, Sadness, Distress, Guilt, Anger, Disgust, Shame) using a Likert-type scale (0 = *‘None’* to 7 = ‘*Strongly*’) and six positive emotion words, not relevant to this investigation. The words used represent a continuous range of arousal/activation within the negative valence of the affective circumplex [81] and are similar to those used in other studies of ED [11]. Ratings were made up to 5 times per day over 14 total days (excluding word-learning task days), for a total of 70 possible prompts for each participant. One prompt each day contained an accuracy check item in which participants were asked to select ‘0’ to ensure they were reading the presented content. Accuracy responses and number of prompts completed were reviewed. Of the whole sample (*N* = 150), five individuals did not provide any diary data. Overall, diary compliance rates were good (Overall: *M* = 55.42 out of 70 (79%), *SD* = 14.35; Pre-task: *M* = 30.15 out of 35 (86%), *SD* = 7.17; Post-task: *M* = 25.28 out of 35 (72%), *SD* = 10.05). In line with established experience sampling procedures [82], five participants (of the 118) who completed less than two SDs below the average number of total diaries (cutoff < 26.96 responses) were excluded in analyses where relevant.

*Emotion differentiation*. Two separate NED scores were generated for each participant from emotion ratings collected pre- and post-word learning task respectively. An index of NED was derived by calculating intraclass correlations coefficients (ICCs) with absolute agreement across the set of negative emotion words. Small correlations suggest that an individual can use more discrete terms to describe their emotional experiences, while large correlations suggest individuals describing their emotional experiences in a more uniformed manner. Scores were reversed by subtracting from 1 to aid interpretation, with larger values corresponding to higher ED [24]. Nine participants were excluded in our analyses because they did not have pre-/post-task NED scores due to limited variation across emotion word usage in a pattern that suggested inaccurate reporting and/or measurement error.

*Negative emotion intensity*. Negative emotion intensity (NEI) was derived by calculating the mean across participants’ ratings of negative emotion words across each 7-day phase (pre- and post-task) experience sampling (see Tables 1 and 2). NEI has been consistently explored in ED research as higher levels of intensity may demand more ED [11].

#### Word learning task engagement

To assess the extent with which participants were engaged in the word learning task across both emotion and neutral conditions, we examined their performance on the memory recall task and multiple-choice quizzes (from both Day one and two).

*Memory recall performance*. Participants’ engagement with the memory task was assessed by coding the specificity of recalled memories associated with each emotion (vs. household item) word. A trained research assistant (blinded to study’s hypotheses) coded each response (range: 1–5) based on whether participants provided a specific memory (5; e.g., “I felt sad when I had to say goodbye to my family during freshman year of college.”), general memory (4; e.g., “I feel sad whenever I miss my friends.”), definition of the emotion (or household item) word (3), unsure (2), or incorrect and/or missing data (1). These scores were then averaged to provide an index of the extent with which participants were able to generalize knowledge acquired through the word learning task on self-relevant memories. The mean score for those in the emotion condition (n = 58) was 4.34 (SD = .41; range: 2.67–5.00) and those in the neutral condition (n = 60) was 4.57 (SD = .38; range: 3.07–5.00).

*Multiple-choice quiz scores*. To examine participants’ quiz performance, we calculated the total number of errors (out of all 15 questions) committed for each quiz taken on Day one and two respectively. Across both conditions, participants did not commit any errors for the quiz taken on Day one. Conversely, participants committed errors for the quiz taken on Day two across both conditions. Specifically, the mean error rate for those in the emotion condition (n = 58) was .06 (SD = .08; range: 0–.27) and those in the neutral condition (n = 60) was .07 (SD = .08; range: 0–.33).

### Data analytic plan

First, we planned to confirm that groups were equivalent at baseline using chi-square and independent samples t-tests on all demographic and pre-task measures. If groups differed on any variables of interest, we included them as covariates in subsequent analyses. We also ran bivariate correlations between baseline and post-task variables to identify relationships between variables and potential covariates to include in subsequent analyses.

To examine the effects of the word learning task (emotion vs. neutral condition) on primary outcomes, we conducted a series of independent samples t-tests to assess group differences in NED, ESE, and aggregate measure of symptoms/distress. If non-significant findings emerged, we conducted equivalence testing using the two one-sided tests (TOST) approach as outlined in Lakens et al. [65] to ascertain whether non-significant results were true null effects or rather due to our sample being underpowered to detect between-group differences. This approach involves setting two equivalence bounds that enables examination of whether we can “reject effects that are as extreme or more extreme than our smallest effect size of interest” (p. 47) [65]. Due to limited prior work, we set equivalence bounds based on small effect size (Cohen’s d = ± 0.2) following convention to be conservative.

**Fig 2:**
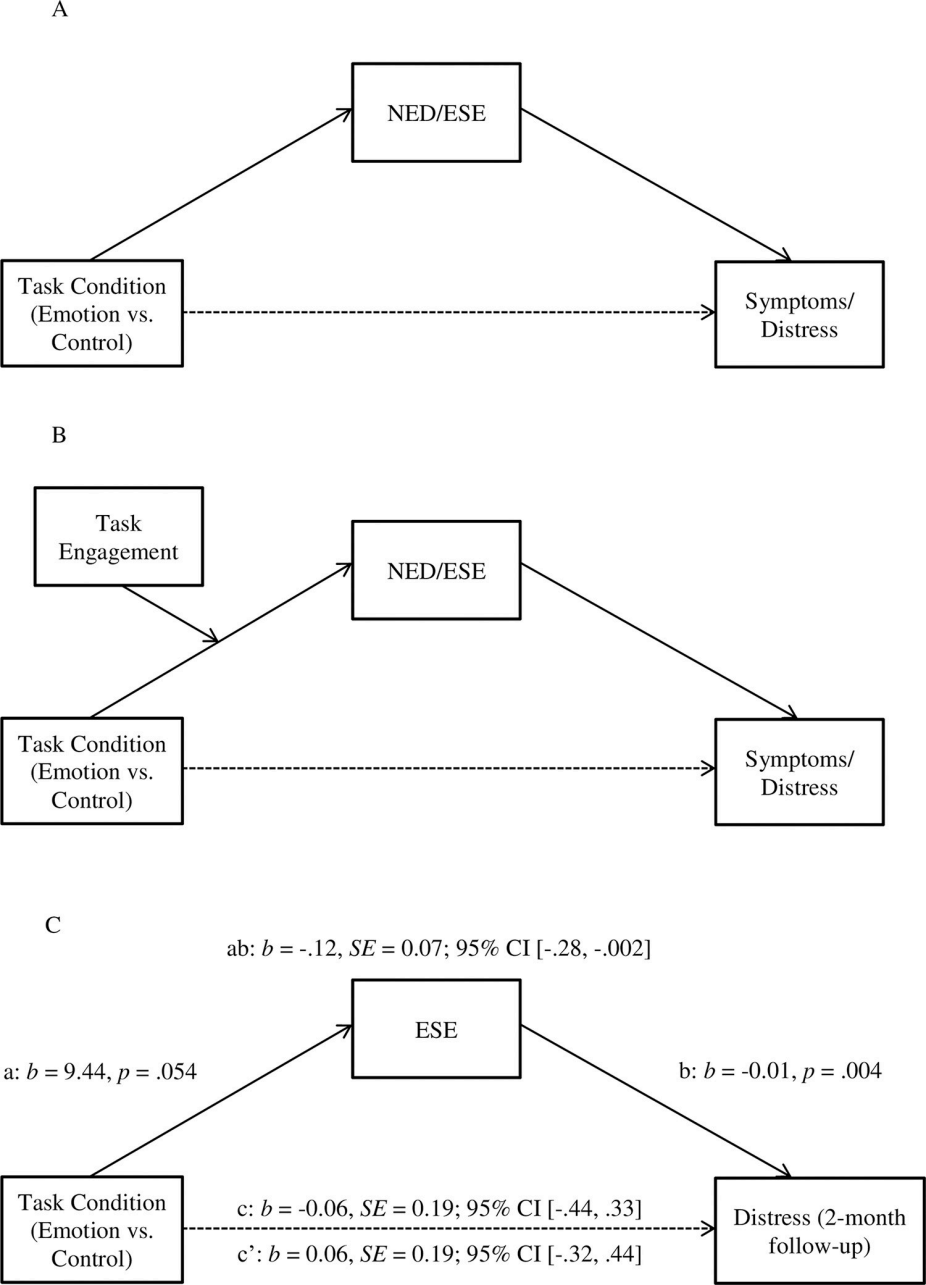
Examples of mediation (2A) and moderated mediation (2B) models in which negative emotion differentiation or emotional self-efficacy mediates the relationship between task condition and post-task symptoms/distress, and whether these associations were moderated by level of task engagement. Fig 2C presents the effect of task condition on symptoms/distress at two-month follow-up as mediated by post-task emotional self-efficacy. The values on the *c* path are for the total effect, *c’* path are for the direct effect, and *ab* path are for the indirect effect. *Note*. NED = Negative Emotion Differentiation; ESE = Emotional Self-Efficacy; emotion condition coded as 1, control condition coded as 0.

Finally, we were interested in understanding whether NED or ESE might underlie the potential relationship between task condition and distress (direct effects), where increases in NED or ESE post-task might result in reductions in distress over time (indirect effects). Therefore, we planned to conduct mediation analyses using Hayes’ PROCESS macro (Model 4) [83] to explore both direct and indirect effects. Further, to explore whether levels of task engagement (as assessed by memory specificity and quiz scores) influenced these associations, we conducted moderated mediation analyses using Hayes’ PROCESS macro (Model 7) [83]. Fig 2 provides an illustration of both mediation (2A) and moderated mediation (2B) models.

## Results

### Preliminary analyses

We first confirmed that there were no differences in participants removed because of poor compliance or failure to meet accuracy checks and those that were maintained. Table 1 describes correlations and descriptive information between baseline and post-task variables for the sample (*n* = 118). We found that the aggregate measure of distress across time points was significantly negatively associated with baseline and post-task ESE, and significantly positively associated with baseline and post-task negative emotion intensity. Baseline NED was significantly negatively associated with post-task distress and at two-month follow-up. As in past research, NED was negatively associated with negative emotion intensity. Baseline verbal intelligence was not associated with any variables of interest. No other significant associations emerged between primary outcomes of interest.

Then, we confirmed that our experimental groups did not meaningfully differ on any key dimension at baseline. Indeed, as described in Table 2, chi-square and independent samples t-tests revealed no significant differences in age, gender, race/ethnicity, verbal intelligence, aggregate distress, ESE, NED, and negative emotion intensity between groups at baseline (*p*s > .05). These findings suggest both groups were generally equivalent at baseline. Further, there were no differences between groups in the average total number of diaries completed.

### Primary analyses

#### Post-task group differences

Separate independent samples t-tests were conducted to examine group differences in post-task NED, ESE, and aggregate measure of symptoms/distress (at one-week and two-month post-task). Results are described in Table 2.

*NED*. Results from independent samples t-tests indicated no significant difference between the emotion (n = 52, M = .49, SD = .27) and neutral (n = 52, M = .42, SD = .24) task condition on post-task NED, t(102) = -1.37, p = .174. Using the TOST procedure, results indicated that the observed effect size (d = -0.27) was not significantly within the equivalent bounds of d = -0.2 and d = 0.2, t(100.62) = -0.38, p = .647, mean difference: -.07, 90% CI [-.15; .01] and were not statistically equivalent to zero.

*ESE*. Results from independent samples t-tests indicated no significant difference between the emotion (n = 54, M = 109.35, SD = 22.79) and neutral (n = 57, M = 104.05, SD = 24.48) task condition on post-task ESE, t(109) = -1.18, p = .241. Results from the TOST procedure indicated that the observed effect size (d = -0.22) was not significantly within the equivalent bounds of d = -0.2 and d = 0.2, t(108.97) = -0.13, p = .550, mean difference: -5.30, 90% CI [-12.74; 2.14] and were not statistically equivalent to zero.

*Distress at one-week post-task*. Results from independent samples t-tests indicated no significant difference between the emotion (n = 54, M = -.11, SD = .86) and neutral (n = 55, M = .12, SD = 1.01) task condition on distress at one-week post-task, t(107) = 1.26, p = .210. Results from the TOST procedure indicated that the observed effect size (d = 0.24) was not significantly within the equivalent bounds of d = -0.2 and d = 0.2, t(104.88) = 0.22, p = .587, mean difference: .23, 90% CI [-0.07; 0.53] and were not statistically equivalent to zero.

*Distress at two-month post-task*. Results from independent samples t-tests indicated no significant difference between the emotion (n = 48, M = -.04, SD = .90) and neutral (n = 46, M = .04, SD = .96) task condition on distress at two-month post-task, t(92) = .41, p = .680. Results from the TOST procedure indicated that the observed effect size (d = 0.09) was not significantly within the equivalent bounds of d = -0.2 and d = 0.2, t(90.93) = -0.56, p = .289, mean difference: 0.08, 90% CI [-0.24; 0.40] and were not statistically equivalent to zero.

Overall, we did not observe significant between-group differences in post-task variables. In addition, results from equivalence tests suggest that we do not have sufficient information to affirm the null hypothesis either. Together, these findings indicate that group differences may be small and that our sample may be underpowered to detect them.

#### Mediation analyses

To examine the direct effects of task condition on distress and NED/ESE, as well as the indirect effects of post-task NED/ESE on the relationship between task condition and distress at one-week and two-month post-task, separate bootstrap mediation analyses using Hayes’ PROCESS macro (Model 4) [83] were conducted using 10,000 bootstrap samples. Group (emotion [coded 1] vs. control [coded 0]) was entered as the independent variable, distress was entered as the dependent variable, and either post-task NED or ESE was entered as the mediator variable (Fig 2A).

*NED*. For the analyses examining post-task NED as a mediator, we also considered covariates such as baseline verbal intelligence and distress, pre-task NED, and post-task NEI but they did not significantly impact the results and are not reported here.

*Distress at one-week post-task (Table 3)*. No significant direct effects were found between group (emotion: n = 50; neutral: n = 52) and post-task NED (*b* = .08, *SE* = 0.05, *p* = .120) or post-task distress (*b* = -.19, *SE* = 0.19, *p* = .333). There was also no direct effect between post-task NED and distress (*b* = -.31, *SE* = 0.37, *p* = .407). Additionally, post-task NED did not significantly mediate the relationship between group and one week post-task distress (*b* = -.03, *SE* = 0.04, 95% CI [-.11, .04]).

**Table 3:** Mediation analysis examining post-task NED as a mediator of the relationship between group and one-week post-task distress. Estimates based on 10,000 bootstrap samples (*n* = 102).

Group as a Predictor of Post-Task NED (*a* path)							
Predictor	*B*	*SE*	*t*	*p*	CI Lower	CI Upper	*R^2^*
Constant	0.42	0.04	11.88	< .001	0.35	0.49	
Group	0.08	0.05	1.57	.120	-0.02	0.18	
*F*(1, 100) = 2.46, *p* = .120							0.02
Post-Task NED (*b* path) and Group (*c* path) as Predictors of Post-Task Distress							
Predictor	*B*	*SE*	*t*	*p*	CI Lower	CI Upper	*** ***
Constant	0.28	0.20	1.39	.167	-0.12	0.69	
Post-Task NED	-0.31	0.37	-0.83	.407	-1.05	0.43	
Group	-0.19	0.19	-0.97	.333	-0.56	0.19	
*F*(2, 99) = 0.97, *p* = .384							0.19
Indirect Effect of Group on Post-Task Distress via Post-Task NED (*ab* path)							
** **	Indirect Effect	*SE*	* *		CI Lower	CI Upper	
Post-Task NED	-0.03	0.04			-0.11	0.04	

*Note*. Distress = Aggregated SCL-90/IDAS-II standardized scores, NED = Negative Emotion Differentiation; emotion condition coded as 1, control condition coded as 0.

*Distress at two-month post-task (Table 4).* As above, there were no significant direct effects found between group (emotion: n = 45; neutral: n = 43) and post-task NED (*b* = .06, *SE* = 0.05, *p* = .261) or two-month post-task distress (*b* = -.11, *SE* = 0.20, *p* = .599). There was also no direct effect between post-task NED and two-month post-task distress (*b* = -.32, *SE* = 0.42, *p* = .447). Similarly, there was no evidence of mediation by post-task NED on two-month post-task distress (*b* = -.02, *SE* = 0.04, 95% CI [-.11, .04]).

**Table 4:** Mediation analysis examining post-task NED as a mediator of the relationship between group and two-month post-task distress. Estimates based on 10,000 bootstrap samples (*n* = 88).

Group as a Predictor of Post-Task NED (*a* path)							
Predictor	*B*	*SE*	*t*	*p*	CI Lower	CI Upper	*R^2^*
Constant	0.42	0.04	11.49	< .001	0.35	0.49	
Group	0.06	0.05	1.13	.261	-0.04	0.16	
*F*(1, 86) = 1.28, *p* = .261							0.01
Post-Task NED (*b* path) and Group (*c* path) as Predictors of Two-Month Post-Task Distress							
Predictor	*B*	*SE*	*t*	*p*	CI Lower	CI Upper	*** ***
Constant	0.22	0.23	0.96	.339	-0.23	0.67	
Post-Task NED	-0.32	0.42	-0.76	.447	-1.14	0.51	
Group	-0.11	0.20	-0.53	.599	-0.50	0.29	
*F*(2, 85) = 0.49, *p* = .616							0.01
Indirect Effect of Group on Two-Month Post-Task Distress via Post-Task NED (*ab* path)							
** **	Indirect Effect	*SE*	* *		CI Lower	CI Upper	
Post-Task NED	-0.02	0.04			-0.11	0.04	

*Note*. Distress = Aggregated SCL-90/IDAS-II standardized scores, NED = Negative Emotion Differentiation; emotion condition coded as 1, control condition coded as 0.

*ESE*. For the analyses examining post-task ESE as a mediator, we also considered covariates such as baseline distress and ESE and reported if they significantly impacted the results.

*Distress at one-week post-task (Table 5)*. No significant direct effects were observed between group (emotion: n = 53; neutral: n = 54) and post-task ESE (*b* = 6.71, *SE* = 4.56, *p* = .144) or post-task distress (*b* = -.12, *SE* = 0.17, *p* = .479). However, there was a direct effect of post-task ESE on one-week post-task distress (*b* = -0.01, *SE* = 0.004, *p* < .001) such that higher ESE was associated with lower levels of distress. As with NED, ESE did not significantly mediate the relationship between group and one-week post-task distress (*b* = -.09, *SE* = 0.07; 95% CI [-.24, .03]).

**Table 5:** Mediation analysis examining post-task ESE as a mediator of the relationship between group and one-week post-task distress. Estimates based on 10,000 bootstrap samples (*n* = 107).

Group as a Predictor of Post-Task ESE (*a* path)							
Predictor	*B*	*SE*	*t*	*p*	CI Lower	CI Upper	*R^2^*
Constant	102.70	3.21	32.03	< .001	96.35	109.06	
Group	6.71	4.56	1.47	.144	-2.32	15.74	
*F*(1, 105) = 2.17, *p* = .144							0.02
Post-Task ESE (*b* path) and Group (*c* path) as Predictors of Post-Task Distress							
Predictor	*B*	*SE*	*t*	*p*	CI Lower	CI Upper	*** ***
Constant	1.49	0.40	3.74	< .001	0.70	2.28	
Post-Task ESE	-0.01	0.00	-0.71	< .001	-0.02	-0.01	
Group	-0.12	0.17	-3.67	.479	-0.47	0.22	
*F*(2, 85) = 0.49, *p* = .616							0.13
Indirect Effect of Group on Post-Task Distress via Post-Task ESE (*ab* path)							
** **	Indirect Effect	*SE*	* *		CI Lower	CI Upper	
Post-Task ESE	-0.09	0.07			-0.24	0.03	

*Note*. Distress = Aggregated SCL-90/IDAS-II standardized scores, ESE = Emotional Self-Efficacy; emotion condition coded as 1, control condition coded as 0.

*Distress at two-month post-task (Table 6).* No significant direct effect was observed between group (emotion: n = 45; neutral: n = 45) and two-month post-task distress (*b* = .06, *SE* = 0.19, *p* = .756). The direct effect of group on post-task ESE was trending towards significance (*b* = 9.44, *SE* = 4.83, *p* = .054). However, there was a direct effect of post-task ESE on two-month post-task distress (*b* = -0.01, *SE* = 0.004, *p* = .004) such that higher ESE was associated with lower levels of distress. Importantly, post-task ESE emerged as a significant mediator of the relationship between group and two-month post-task distress, such that higher ESE in those in the emotion condition were related to lower levels of distress at two months (*b* = -.12, *SE* = 0.07; 95% CI [-.28, -.002]). These results are illustrated in Fig 2C and detailed in Table 6. Note that the mediation effect disappeared after accounting for baseline distress and ESE.

**Table 6:** Mediation analysis examining post-task ESE as a mediator of the relationship between group and two-month post-task distress (controlling for baseline ESE and distress). Estimates based on 10,000 bootstrap samples (*n* = 90).

Group as a Predictor of Post-Task ESE (*a* path)		
Predictor	*B*	*SE*
Constant	46.95	14.38
Baseline Distress	-0.86	2.74
Baseline ESE	0.52	0.13
Group	6.56	4.44
*F*(3, 86) = 8.71, *p* < .001		
Post-Task ESE (*b* path) and Group (*c* path) as Predictors of Two-Month Post-Task Distress		
Predictor	*B*	*SE*
Constant	0.24	0.52
Baseline Distress	0.63	0.09
Baseline ESE	0.00	0.01
Post-Task ESE	-0.01	0.00
Group	0.19	0.15
*F*(4, 85) = 15.98, *p* < .001		
Indirect Effect of Group on Two-Month Post-Task Distress via Post-Task ESE (*ab* path)		
** **	Indirect Effect	*SE*
Post-Task ESE	-0.05	0.07

*Note*. Distress = Aggregated SCL-90/IDAS-II standardized scores, ESE = Emotional Self-Efficacy; Emotion condition coded as 1, control condition coded as 0.

#### Moderated mediation analyses

To explore participants’ level of engagement in the intervention itself (i.e., memory specificity and quiz error rate) as a moderator in the mediation model, separate bootstrap moderated mediation analyses using Hayes’ PROCESS macro (Model 7) [83] were conducted using 10,000 bootstrap samples. Group (emotion [coded 1] vs. control [coded 0]) was entered as the independent variable, distress was entered as the dependent variable, either post-task NED or ESE was entered as the mediator variable, and either memory specificity or quiz error rate was entered as the moderator variable (Fig 2B). As above, we considered baseline distress and pre-task NED/ESE as potential covariates and reported if they significantly impacted the results.

*Memory specificity as moderator*. We found significant moderated mediation effects for the model with post-task NED as the mediator of the relationship between group and one-week post-task distress, controlling for baseline distress and pre-task NED as covariates (n = 100). These effects are described in Table 7. Specifically, memory specificity during the intervention itself significantly moderated the association between group and post-task NED (b = .35, SE = .13, p = .011). Simple slopes analysis indicated that among those in the emotion condition, greater memory specificity (+1SD) was associated with higher levels of post-task NED (b = .20, SE = .07, p = .006) but this association was not observed among those at mean (b = .06, SE = .05, p = .241) and lower (-1SD) memory specificity (b = -.08, SE = .08, p = .320). Importantly, the results indicated that post-task NED significantly mediated the relationship between group and one-week post-task distress among individuals demonstrating higher memory specificity (or task engagement) in the emotion condition, b = -.10, SE = .06, 95% CI [-.22, -.01]. No significant mediation effects were observed among those at mean or lower memory specificity.

**Table 7:** Moderated mediation results for conditional indirect effects (*n* = 100).

Group and Task Engagement (Memory Specificity) Interaction as a Predictor of Post-Task NED (*a* path)							
Predictor	*B*	*SE*	*t*	*p*	CI Lower	CI Upper	*R^2^*
Constant	0.35	0.06	6.06	< .001	0.24	0.47	
Baseline NED	0.23	0.10	2.25	.027	0.03	0.43	
Baseline Distress	0.01	0.03	0.21	.833	-0.05	0.06	
Group	0.06	0.05	1.18	.241	-0.04	0.16	
Memory Specificity	-0.23	0.10	-2.24	.028	-0.44	-0.03	
Group × Memory Specificity	0.35	0.13	2.61	.011	0.08	0.61	
*F*(5, 94) = 2.86, p = .019							0.13
Conditional Effect of Group on Post-Task NED at High/Moderate/Low Levels of Task Engagement							
** **	*b*	*SE*	*t*	*p*	CI Lower	CI Upper	
Low Task Engagement (-1 SD)	-0.08	0.08	-1.00	.320	-0.23	0.08	
Moderate Task Engagement (M)	0.06	0.05	1.18	.241	-0.04	0.16	
High Task Engagement (+1 SD)	0.20	0.07	2.83	.006	0.06	0.34	
Post-Task NED (*b* path) and Group (*c* path) as Predictors of Post-Task Distress							
Predictor	*B*	*SE*	*t*	*p*	CI Lower	CI Upper	*R^2^*
Constant	0.52	0.17	3.06	.003	0.18	0.86	
Baseline NED	-0.52	0.27	-1.95	.054	-1.05	0.01	
Baseline Distress	0.72	0.07	10.58	< .001	0.58	0.85	
Post-Task NED	-0.51	0.26	-1.99	.049	-1.03	-0.001	
Group	0.03	0.13	0.2	.840	-0.23	0.28	
*F*(4, 95) = 32.87, p < .001							0.58
Conditional Indirect Effect of Group on Post-Task Distress via Post-task NED at Levels of Task Engagement							
** **	*b*	*SE*	* *	* *	CI Lower	CI Upper	** **
Low Task Engagement (-1 SD)	0.04	0.04			-0.05	0.13	
Moderate Task Engagement (M)	-0.03	0.03			-0.11	0.02	
High Task Engagement (+1 SD)	-0.10	0.06			-0.22	-0.01	

*Note*. Distress = Aggregated SCL-90/IDAS-II standardized scores; NED = Negative Emotion Differentiation; emotion condition coded as 1; control condition coded as 0.

*Quiz performance as moderator*. No significant findings emerged across all mediation models testing quiz error rate as a moderator, even after controlling for baseline distress and pre-task NED/ESE. Specifically, the indirect effect of group on distress (at one-week or two-month post-task) through post-task NED/ESE did not vary as a function of quiz error rate.

## Discussion

The current study examined the effects of a novel brief online emotion word learning task on NED, ESE, and distress in a college sample. NED and ESE were examined as potential mediators of the relationship between word learning task condition and distress. Finally, we also explored whether the strength of these associations was influenced by participants’ level of engagement with the word learning task. Our key findings indicate that a relatively simple and straightforward emotion language learning task can influence short and long term outcomes through increases in emotional self-efficacy and shifts in NED, when participants are well-engaged. These findings have clear implications for the development of novel interventions targeting underlying dimensions of disrupted transdiagnostic emotional processes like NED and ESE.

Overall, our findings provide partial support for the efficacy of the emotion word learning task on primary outcomes of interest. While we did not observe significant group differences in NED, ESE, and distress post-task (and at two months), however, post-hoc tests of equivalence suggest that these null findings were not statistically equivalent to zero either. Taken together, these results indicate that the efficacy of our task remains preliminary and would benefit from future replication, preferably in larger well-powered samples. Despite the lack of significant group differences in outcomes, we did observe significant indirect effects of post-task ESE on distress at two months follow-up, where increases in ESE among participants in the emotion word learning condition were related to lower levels of distress. This suggests that individuals in the experimental condition who experienced gains in ESE had some long-term psychological benefits despite the brief nature of the word learning task. Finally, we found indirect effects of post-task NED on distress at one-week post-task that depended on the extent with which participants engaged in the word learning task. Specifically, the emotion word learning task predicted increases in post-task NED among those who demonstrated higher task engagement (as indicated by greater specificity of emotional memory recalled), which in turn predicted lower levels of distress at one-week post-task. No other significant findings were observed.

Together these preliminary results suggest the possibility that a relatively simple emotion word learning task could influence how individuals attend to negative emotional experiences in daily life. Although there was no evidence of direct improvement in NED or ESE, there were some evidence of indirect short-term and long-term effects on psychological health that was mediated by increases in NED (depending on level of task engagement) and ESE respectively. These preliminary findings provide an exciting starting point for further research given the simplicity of the intervention and the potential for clinically meaningful impact on common treatment targets.

### Emotion differentiation as a mediator

Critically, our results also demonstrate the importance of task engagement. Specifically, we found some evidence suggesting that the intervention was able to indirectly impact short-term distress/psychological health through post-task NED. However, this mediation was dependent on level of engagement in the intervention. Indeed, when individuals were more able to apply emotion knowledge to generate a specific personal memory, they were more likely to demonstrate improved differentiation abilities that in turn predicted lower distress one-week later. That NED emerged as a significant mediator between the emotion word learning task and post-task distress (among highly engaged participants) is in line with prior intervention research. For instance, Van der Gucht et al. [34] found that participants demonstrated improvements in NED following an 8-week mindfulness training while Vedernikova et al. [33] observed similar effects from a 5-day emotion knowledge intervention. Taken together, these findings suggest that exposure to interventions that cultivate emotional awareness and emotion word learning may be a potentially meaningful way to positively influence the relationship between ED and psychological health. Notably, our online word learning intervention was brief and, despite the demands, had excellent compliance and completion rates from participants. Therefore, it may be a promising basis for the development of a future low-cost and scalable transdiagnostic treatment to consider.

The finding that participants’ scores on the emotion knowledge multiple choice quiz did not impact differentiation abilities is largely in line with both learning science [68] and contemporary treatments such as CBT and DBT which focuses on not just skills/knowledge *acquisition* but also *application* and/or *generalization* of these skills/knowledge across contexts [35,84]. Moreover, this finding suggests that perhaps more attention should be devoted to scaffolding patients’ ability to apply emotion concepts to personally relevant experiences in treatment to maximize benefits. For example, the current intervention might have been enhanced if participants were asked to generate another personal memory the next day. Finally, that task engagement was important also suggests an important avenue to explore in future treatment research and development.

### Emotional self-efficacy as mediator

Unlike NED, we found that higher ESE was significantly associated with lower levels of distress not only concurrently but also two months post-task. Importantly, ESE was found to significantly mediate the relationship between task condition and distress at two-month follow-up, with higher ESE among those in the emotion word learning condition demonstrating prospective reductions in distress. These findings bring forth the interesting possibility that feelings of competency around emotions, perhaps more so than specific emotion skills, may be important in predicting psychological health and related outcomes. Indeed, these findings are in line with decades of research on self-efficacy, which found that individuals’ perceived competency was predictive of a wide range of behavioral and clinical outcomes, above and beyond that of their actual abilities [54,85,86]. Given its benefits, future research should continue to explore additional ways to enhance ESE, as well as how ESE may impact other related constructs important for psychological health.

That ESE was associated with long-term psychological benefits in the current study also suggests that it may be an important and relatively simple target to consider in treatment development. This may be particularly relevant among patients experiencing high intensity negative emotions and difficulties with emotion regulation, both of which feature prominently across numerous psychological disorders [1]. Already, treatments such as DBT have included skills focused on increasing individuals’ ability to manage their emotions by building a sense of mastery/self-efficacy [35]. Moreover, interventions such as exposure therapy for anxiety-related disorders that focus on systematically increasing patients’ ability to tolerate feared stimuli have also been shown to influence levels of self-efficacy [87,88]. Indeed, rigorous experimental investigations have demonstrated that instructing individuals to label their affect during exposure can dampen physiological arousal, as well as increase willingness to approach feared stimuli at follow-up despite no apparent reductions in self-reported fear [31,89]. Together, these findings suggest that attention to changes in ESE could be a useful target in treatment research, one that can be explicitly and easily measured and monitored in relation to treatment progress in the clinic.

### Strengths and limitations

In reviewing the results of the current study, it is important to do so in the context of its strengths and limitations. Notable strengths include the explicit focus on an underlying dimension of emotion processing (rather than a symptom specific to a disorder) assessed using robust methods including intensive sampling of emotions experienced in daily life via semi-random experience sampling. This is considered the gold standard of mood measurement [90] as it allows for assessment during the course of everyday life (vs. in the laboratory) and is minimally influenced by memory and other affective distortion biases [91–93]. An additional strength includes the effort to isolate the effects of the task by including an active, well-matched, control task and focusing on explicit investigation of alternate processes, including both the development of perceived mastery via measurement of emotional self-efficacy and the assessment of engagement with the intervention itself.

Weaknesses include a relatively limited retrieval activity for the active intervention. The learning literature suggests that multiple opportunities for retrieval of new content [72] along with delayed feedback [94] can both enhance memory and recall. A slightly longer task may be able to make effective use of these techniques to generate more durable prospective changes in NED, ESE, and symptoms. However, our focus here was on the practicality of a brief, simple, and remotely-administered intervention. Undoubtedly, the duration and intensity of retrieval could be extended and tested in replication to maximize any benefits. In addition, unlike the multiple-choice quiz, we did not assess pre-post changes in memory recall performance. Therefore, it is unclear whether improvements in NED were driven by task performance or that participant-level factors (e.g., conscientiousness) contributed to these changes. This will be important to delineate in future research by including additional pre-post tests of task engagement. In addition, our findings show that while more NED and ESE appear to be associated with less distress, task condition accounted for a relatively small amount of variance in our models. This suggests that emotion word learning is one piece of the puzzle when it comes to enhancing emotion vocabulary and competency, and that exploration of other techniques to increase these important emotion skills is warranted. Finally, past research utilizing experience sampling has found that just the act of monitoring one’s emotions over time may enhance awareness and improve differentiation abilities [62,95]. Combined with the relatively brief nature of our intervention and limited sample size, this may explain the lack of direct effects on primary outcomes of interest. Indeed, emotion conceptualization processes are largely formed over years during childhood [96,97] and a brief intervention may not be sufficient for lasting change in some adults.

### Clinical implications and future directions

Despite limitations, our preliminary findings have important implications for treatment development. Notably, we observed indirect effects of NED and ESE on psychological health. These findings are largely consistent with existing research on NED and ESE, and suggest that both NED and ESE may be important and relatively straightforward psychological mechanisms to consider in treatment. While facilitating emotion language use and mastery are common across psychotherapies, this is done with varied intention [10]. Our findings suggest that more explicit focus on these affective processes in treatment may be helpful in improving psychological outcomes, which is consistent with prior research demonstrating how affect labeling may enhance effectiveness of exposure therapy [98]. Indeed, the present study utilized a dismantling approach that focused on isolating the effects of emotion word learning on NED, ESE, and psychological health. Although preliminary, these results provide a starting point for informing the development of novel interventions further aimed at addressing key transdiagnostic emotional processes.

In the future, our task would benefit from replication in larger and more diverse samples beyond college students, including patient populations where we would expect greater variability in affective processes and psychological symptoms/distress. In doing so, focus should be placed on further elucidating the mechanism(s) by which emotion labeling comes to be beneficial and relevant individual factors that determine for who and when it is appropriate to offer such a task through rigorous experimental work.

## Conclusions

The current study examined the effectiveness of a brief online emotion word learning task to determine whether it impacted key processes linked to emotion-related disorders, namely ED and ESE, and consequently tested if reduced psychological distress in the short and long-term. The results suggest that with good engagement in the training, emotion word learning may be one way to enhance important potential transdiagnostic processes such as ED and ESE and reduce distress in the short-term and long-term respectively. Moreover, our findings suggest that this task can be effectively delivered remotely without the aid of a trained mental health provider. Future work should seek to replicate and better understand the mechanisms underlying emotion labeling, its relationship with ED and ESE, and consider the further development of emotion word learning interventions as potential adjuvant tools to be implemented in clinical settings.
